# SCRS: Segment Structure with Controllable Realistic Synthetic for Chip Scratch Detection

**DOI:** 10.3390/s25226868

**Published:** 2025-11-10

**Authors:** Jiaqing Huang, Jianjun He, Weihua Gui

**Affiliations:** School of Automation, Central South University, Changsha 410083, China; alyerh@csu.edu.cn (J.H.); gwh@csu.edu.cn (W.G.)

**Keywords:** laser diode chip, data synthesis, diffusion model, scratch segmentation

## Abstract

This paper proposes the Segment Structure with Controllable Realistic Synthetic (SCRS) to address the challenge of detecting scratches on laser diode chip emitting facets, which can impair laser emitting and cause chip burnout. Scratch detection is critical for ensuring laser quality and stability, but low-contrast images hinder comprehensive dataset creation. SCRS leverages a mask-guided diffusion model to generate diverse, realistic synthetic scratch images, enabling robust training data synthesis. The generated dataset trains a novel TransCNN network, which combines vision transformer blocks and convolutional decoding for accurate scratch segmentation. Experimental results show that SCRS achieves mean Intersection over Union (mIoU) values of 74.4% for deep scratches and 75.8% for shallow scratches, demonstrating its significant potential for industrial applications.

## 1. Introduction

The laser diode chip, a fundamental component of laser systems, is distinguished by its compact size and high power output, enabling its widespread application in the fields of optical communication and medical and industrial manufacturing [1]. However, its small dimensions make the emitting facet highly prone to pronounced and subtle scratches during various manufacturing processes [2]. Such scratches can compromise the integrity of the chip and, in severe cases, cause laser energy to concentrate at defect sites, resulting in chip burnout. Therefore, detecting scratches on the emitting facet is essential for ensuring laser quality and operational reliability.

**Figure 1:**
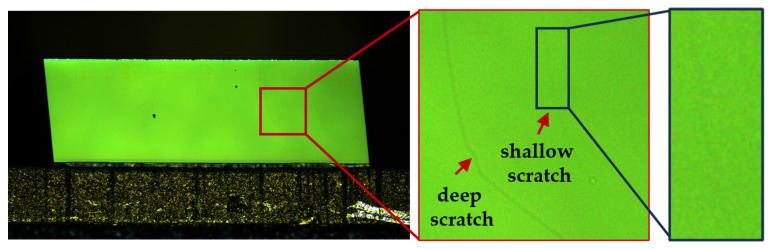
Scratches on the emitting facet.

The semiconductor industry predominantly employs image semantic segmentation to detect scratches on laser diode chips. As illustrated in Figure 1, an image of a chip’s emitting facet under 20× magnification reveals a surface with approximate dimensions of 2 mm × 0.14 mm. The magnified region in Figure 1 contains a deep scratch and a shallow scratch, each with a width of less than 10 pixels in the image, corresponding to sub-micrometer scales in reality. Deep scratches exhibit a high contrast against the background, facilitating their identification. In contrast, shallow scratches closely resemble the background, making them challenging to detect even with human vision. Such subtle scratches constitute approximately 20% of all scratch-related defects. In addition, the high noise levels introduced by high-magnification imaging further exacerbate the difficulty of identifying these shallow scratches.

Scratch segmentation mainly involves traditional methods and deep learning techniques [3]. Traditional approaches often utilize topology and mathematical modeling. Peng et al. proposed an enhanced watershed algorithm with optimal labeling and edge constraints, using edge operators to refine boundaries for efficient segmentation of low-contrast, high-noise foam images [4]. Kishorjit et al. combined simple linear iterative clustering superpixels with an adaptive Gaussian radial basis function kernel-based fuzzy C-means method to improve segmentation robustness [5]. Cai et al. developed an adaptive variational level set model, integrating scale bias correction and denoising terms to improve noisy image segmentation [6]. Although traditional methods are computationally efficient and fast, they struggle to detect scratches on the emitting facet due to high noise and indistinct features.

Deep learning approaches for scratch segmentation are data-driven, learning class features from datasets, and categorized into supervised, semi-supervised, and self-supervised methods. Supervised methods utilize labeled datasets to train models capable of segmenting unseen data. Long et al. introduced fully convolutional networks (FCNs) for pixel-level segmentation, outperforming earlier region-classification approaches and enabling industrial inspection applications [7]. Wang et al. proposed FCN-SFW, combining structured forests with wavelet transforms to detect minute cracks in steel beams [8]. U-Net, which extends FCN with an encoder–decoder architecture and skip connections, captures both low- and high-level semantics for biomedical image segmentation [9]. Li et al. improved U-Net with VGG16 and a hybrid attention mechanism for real-time PCB soldering defect detection [10]. Wang et al. further improved U-Net with extended and offset convolutions for SAR image segmentation in aquaculture monitoring [11]. Attention mechanisms have further improved segmentation performance [12]. Yeung et al. proposed ABFormer, which employs a boundary-aware module and attention mechanisms to improve the accuracy of defect segmentation [13]. Although supervised methods excel with sufficient labeled data, annotating shallow scratches on chips is challenging, and models trained on deep scratches often fail to generalize to shallow ones.

Semi-supervised methods utilize limited labeled data combined with techniques such as data augmentation and consistency regularization to learn semantic features from unlabeled data [14]. Shi et al. enhanced pseudo-label reliability in semi-supervised learning through a dynamic threshold strategy, improving defect segmentation accuracy [15]. Chen et al. proposed cross pseudo supervision, where two differently initialized networks mutually guide each other with pseudo-labels to improve consistency and segmentation performance [16]. Zhang et al. developed a weakly supervised method that generates pixel-level annotations from image-level labels, integrating class activation maps with a dense energy loss function to optimize segmentation [17]. Although semi-supervised methods effectively leverage unlabeled data through regularization, their performance may degrade when labeled and unlabeled data differ significantly.

Unsupervised segmentation often employs clustering techniques [18] but struggles to segment scratches in chip images due to their subtle contrast with the background. Self-supervised learning through synthetic data generation provides an alternative. Li et al. used CutPaste data augmentation for self-supervised representation learning, creating a generative one-class classifier for annotation-free defect localization through expanded patch representations [19]. Schlüter et al. utilized Poisson image editing to fuse multi-scale image patches, producing realistic synthetic anomalies to train models for robust defect segmentation [20]. Advances in generative models have enhanced this approach. Zhang et al. introduced Strength-controllable Diffusion Anomaly Synthesis (SDAS), employing diffusion models to generate anomaly patterns superimposed on normal images, which enabled a residual detection model to achieve state-of-the-art defect segmentation [21]. However, reliance on pattern splicing limits the generation of realistic data, impeding effective scratch segmentation in real-world environments.

In summary, the scratch detection task for laser diode chips encounters two main challenges: (1) Annotating shallow scratches is challenging, with current synthesis methods offering insufficient realism and control. (2) The subtle features of shallow scratches, combined with their low contrast against the background, place stringent requirements on the detection model. To address the above challenges, we propose the Segment Structure with Controllable Realistic Synthetic (SCRS). Drawing on extensive analysis of scratch images and their high similarity to normal patterns, we introduce Mask-Guided Local Mean-Shift Diffusion Data Synthesis (MSDS). This method achieves realistic and diverse scratch images through direct generation and mask-based depth control. To address the difficulty of distinguishing scratch patterns from normal ones due to their high similarity, we propose TransCNN, a model that employs ViT blocks for global feature encoding, enhances pattern differentiation through attention mechanisms, and extracts distinct scratch features. Skip connections and convolutional decoding further refine spatial features, improving scratch segmentation accuracy. Experimental results demonstrate that SCRS achieves mean Intersection over Union (mIoU) values of 74.4% for deep scratches and 75.8% for shallow scratches in production, highlighting its significant industrial application value.

## 2. Method

**Figure 2:**
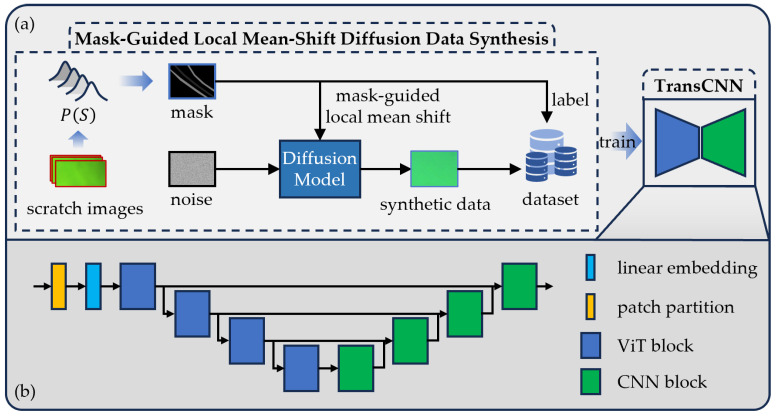
Framework of the SCRS. (**a**) Workflow of the SCRS. (**b**) Architecture of the TransCNN network.

As shown in Figure 2a, SCRS uses MSDS to sample masks. These masks then guide the diffusion model to generate corresponding scratch images for training the TransCNN segmentation network, whose architecture is depicted in Figure 2b. The implementation details of MSDS and TransCNN are described below.

### 2.1. Mask-Guided Local Mean-Shift Diffusion Data Synthesis

Exploiting the similarity and low contrast between scratches and backgrounds in chip images, the Mask-Guided Synthetic Data Synthesis (MSDS) method uses masks to guide diffusion models in performing local mean shift on normal samples, controllably generating synthetic scratch images with varying depths that closely mimic real-world scratches. This approach constructs a robust scratch dataset for training detection models.

#### 2.1.1. Mask Generation

MSDS uses masks to guide diffusion models in generating scratch images, constructing a chip scratch dataset for training detection models. To ensure that the model detects scratches effectively in real-world production, the generated scratch images must align with the actual scratch patterns. Thus, scratches in the masks should accurately reflect those observed in production environments.

This paper characterizes scratches in images using the following metrics: *n*, the number of scratches, $b\in R^{n}$, the width of the individual scratch, $h\in R^{n}$, the depth of the scratch, and *s*, the shape of the scratch. The scratch depth is represented by the maximum pixel contrast between the scratched area and its surrounding region. Scratch shapes are approximated using polygonal chains, with $n_{s}$ being the number of segments used for description. Consequently, the scratch condition in an image can be described by the quadruple $S=\left\{n,b,h,n_{s}\right\}$.

To this end, we randomly sampled chip images acquired from a production line and statistically analyzed the number of scratches (*n*), scratch width (*b*), scratch depth (*h*), and the number of line segments comprising each scratch ($n_{s}$). Based on the law of large numbers, we fitted the distribution of scratch characteristics, *S*, as $P\left(S\right)=P\left(n,b,h,n_{s}\right)$. By sampling the characteristics of scratches for the mask, $p\left(S_{m}\right)$, from $P\left(S\right)$, we can obtain masks that conform to the actual distribution of scratches observed in production, guiding the generation of data that reflect realistic scratch conditions.

Furthermore, we observed that scratches in the images typically exhibit a profile characterized by a deeper central region and shallower edges. Consequently, to ensure that the scratch images generated under mask guidance more closely resemble authentic scratches, we implemented a scratch fine-tuning process on the sampled masks. For a single scratch in the image, given a horizontal scratch center position *c*, a scratch width *b*, and a sampled scratch depth *h*, we fit a Gaussian curve to model the scratch depth profile across its width, as shown in Equation (1).

(1) $$d\left(x\right)=h\cdot w\left(x-c\right)\cdot e^{-\frac{{\left(x-c\right)}^{2}}{2\sigma^{2}}},$$

(2) $$w\left(x\right)=\left\{\begin{array}{c} 1,\left|x\right|\leq \frac{b}{2}\\ 0,\left|x\right|>\frac{b}{2} \end{array}\right.,$$

where $w\left(\cdot \right)$ represents the rectangular window function defined in Equation (2), and σ denotes the standard deviation that governs the width of the scratch. Typically, the scratch depth is set to α·h, where α is a small-edge-depth coefficient. In this case, $\sigma =\frac{b}{\sqrt{8\ln\alpha }}$. Equation (1) is then employed to refine each scratch within the mask, ensuring that the depth variation more closely mirrors that of actual scratches.

#### 2.1.2. Local Mean-Shift Diffusion

**Figure 3:**
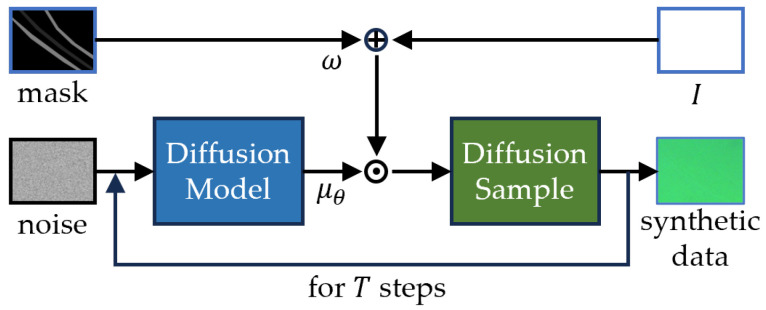
Schematic of the MSDS sampling process, here ⨁ and ⨀ represent element-wise addition and multiplication, respectively.

The MSDS schematic is shown in Figure 3. Built on Denoising Diffusion Probabilistic Models (DDPMs), MSDS trains the model with scratch-free data and uses masks to guide mean-shift operations, generating natural scratches in masked regions.

DDPMs establish a forward diffusion process by progressively introducing Gaussian noise $\varepsilon ∼N\left(0,I\right)$ of increasing intensity to the original data $x_{0}$. At timestep *t*, the conditional probability distribution of $x_{t}$ is given by $q\left(x_{t}\left|x_{t-1}\right.\right)=N\left(x_{t};\sqrt{1-\beta_{t}}x_{t-1},\beta_{t}I\right)$, where ${\left\{\beta_{t}\right\}}_{t=1}^{T}$ represents a fixed, monotonically increasing noise schedule, and ${\left\{x_{t}\right\}}_{t=1}^{T}$ are latent variables. This diffusion process can be described by a Markov chain $q\left(x_{1:T}\left|x_{0}\right.\right)=\prod_{t=1}^{T}q\left(x_{t}\left|x_{t-1}\right.\right)$. Furthermore, the latent variable at time *t* can be sampled directly from $x_{0}$ using the conditional probability distribution $p\left(x_{t}\left|x_{0}\right.\right)=N\left(x_{t};\sqrt{{\bar{\alpha }}_{t}}x_{0},\left(1-{\bar{\alpha }}_{t}\right)I\right)$, where $\alpha_{t}=1-\beta_{t}$ and ${\bar{\alpha }}_{t}=\prod_{i=1}^{t}\alpha_{i}$.

The reverse process employs a deep learning network, parameterized by θ, to learn the reverse sampling distribution $p_{\theta }\left(x_{t-1}\left|x_{t}\right.\right)=N\left(x_{t-1};\mu_{\theta }\left(x_{t},t\right),\Sigma_{t}I\right)$. This leverages a Markov chain to progressively reconstruct $x_{0}$ from $x_{t}$. To model $p_{\theta }\left(x_{t-1}\left|x_{t}\right.\right)$ with a neural network, diffusion models utilize maximum log-likelihood estimation, resulting in the simplified training objective shown in Equation (3). The mean of the reverse-process sampling distribution is then given by μθxt,t=11αtαtxt−βtβt1−α¯t1−α¯tεθxt,t.

(3) $$L_{simple}=E_{t,x_{0},\varepsilon }\left[{∥\varepsilon -\varepsilon_{\theta }\left(x_{t},t\right)∥}^{2}\right].$$

Here, *M* represents the generated mask, ω is a parameter controlling the scratch depth, and ⊙ denotes the pixel-wise product. MSDS leverages the mask *M* to guide the mean-shift region, applying a shift operation to the masked area during sampling. This process yields scratch patterns within the masked region, resulting in labeled data pairs that accurately reflect the distribution of real-world scratches.

Given the high similarity between the scratches and the background in the chip image, we can assume that the image distributions of the scratched and background regions are similar, differing only by a slight shift in their mean values. Therefore, by locally adjusting the mean, we can extract the scratch pattern in this region and generate a chip image with scratches. MSDS performs the sampling process as shown in Equation (4).

(4) $$p_{\theta }\left(x_{t-1}\left|x_{t}\right.\right)=N\left(x_{t-1};\left(I+\omega M\right)⊙\mu_{\theta }\left(x_{t},t\right),\Sigma_{t}I\right),$$

### 2.2. Transcnn for Scratch Detection

With MSDS, we generate a scratch dataset that aligns with real-world scratch characteristics, particularly the low contrast between scratches and backgrounds. To distinguish deep and shallow scratches from the background, we propose TransCNN, as shown in Figure 2b. This model extracts global features, enhances distinctions between deep scratches, shallow scratches, and the background, and refines these features locally to achieve precise scratch segmentation from a global-to-local perspective.

TransCNN employs a patch partition module to decompose the image into HWHWP2P2 non-overlapping patches, where *H* and *W* represent the image’s height and width, respectively, and *P* denotes the patch size. These patches are then encoded into image tokens via a linear layer. The variance between patches with a normal pattern and scratch pattern is further amplified by the attention mechanism in the ViT encoding block. Consequently, after passing through multiple Transformer blocks, the image tokens yield more pronounced scratch features.

The multi-scale features extracted from the ViT blocks are fed into subsequent CNN blocks for localized refinement. Features from different ViT block levels are concatenated with the preceding level’s features and then input to the CNN blocks. These blocks perform convolution, batch normalization, and upsampling operations to achieve local optimization and ultimately generate the final scratch detection result.

## 3. Experiment

SCRS constructs a realistic scratch dataset using MSDS to train TransCNN for the chip scratch segmentation. To validate the feasibility of this framework, we conducted the experiments below.

### 3.1. Dataset

We propose the SCRS: The first part employs the MSDS to generate a training dataset for scratch segmentation, while the second part trains TransCNN for scratch segmentation. The diffusion model in MSDS is trained using normal data. The effectiveness of MSDS is assessed through segmentation performance. Similarly, TransCNN is trained on the MSDS-generated dataset, and its effectiveness is evaluated using segmentation results of real-world scratches. To validate the SCRS framework, we utilize chip images collected from a production line to construct the MSDS training set and the TransCNN test set.

Given that scratches occupy a small proportion of chip images, directly using full images increases computational costs and exacerbates data imbalance, impairing model training. To address this, cropping is adopted during dataset construction, generating images of size 256 × 256 to increase the proportion of scratch regions and mitigate class imbalance.

**Training set.** We cropped 3600 normal images from scratch-free chip images to train the diffusion model in the MSDS. Additionally, we sampled $n_{m}$ masks to generate corresponding scratch images using MSDS, creating a dataset of $n_{m}$ pairs of scratch images. As the distinction between deep and shallow scratches in masks provides limited benefit for detection, we unified all scratches into a single category, further mitigating class imbalance and enhancing training performance.

**Figure 4:**
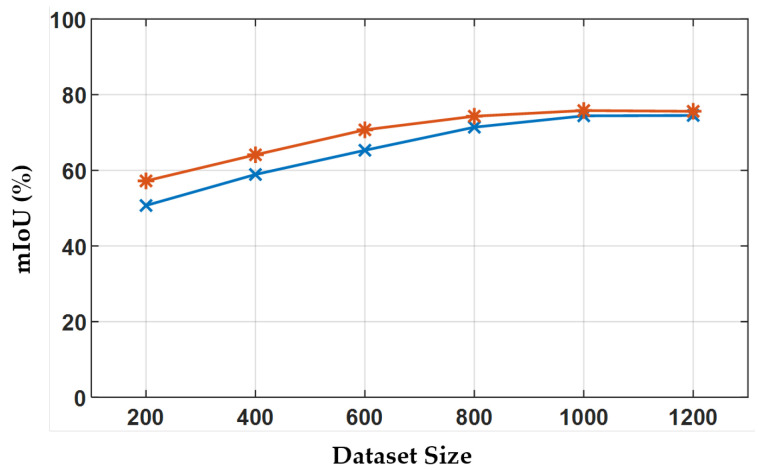
Performance of TransCNN trained on datasets of varying sizes.

To determine an optimal dataset size for scratch training, we generated datasets containing 200, 400, 600, 800, 1000, and 1200 samples and assessed their respective training outcomes, as presented in Figure 4. The segmentation accuracy for deep and shallow scratches enhances with larger datasets, but this effect plateaus beyond a size of 800. Consequently, we adopted a dataset size of $n_{s}=1000$.

**Test set.** We selected 50 scratch-containing images from a production line for meticulous annotation and sampled 300 images with varying scratch depths through random cropping to form the test dataset. Scratches in the test set are qualitatively classified as deep or shallow to evaluate the effectiveness of different methods on varying scratch category.

### 3.2. Implementation Details

All experiments were conducted on a high-performance server, which was equipped with an Intel(R) Xeon(R) Platinum 8368Q CPU @ 2.60 GHz and an NVIDIA A100 GPU with 80 GB of VRAM.

**Figure 5:**
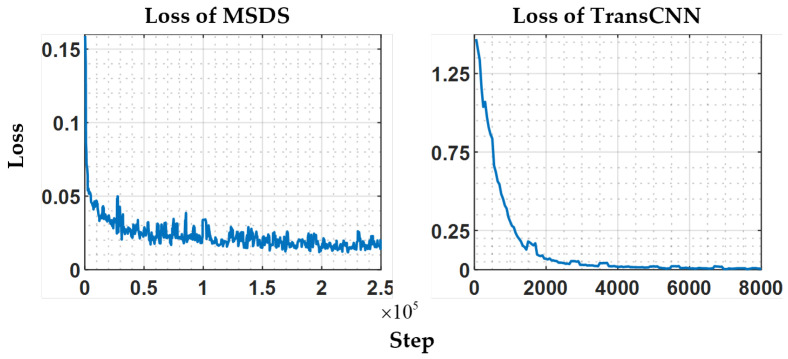
Loss curves of MSDS and TransCNN.

The MSDS employs the U-Net model as the base for its diffusion model, with a diffusion timestep of 1000. The model has 49.8M trainable parameters and achieves a loss convergence to 0.015. TransCNN, with 60.8M trainable parameters, converges to a loss of 0.003. The training loss curves is presented in Figure 5.

The hyperparameter ω controls the degree of mean shift in generating scratch images. Experiments indicate that a small ω results in scratches too similar to normal images, lacking distinct features, while a large ω causes severe distortion and unnatural transitions between scratches and background. ω was set to 0.05 following experimentation.

### 3.3. Msds Sample Results

**Figure 6:**
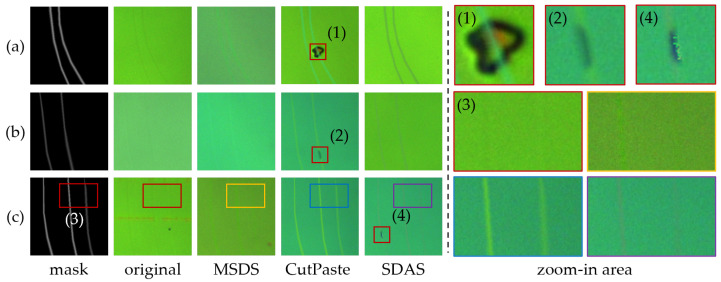
Comparison of scratch image sampling results. (**a**) Mask with only deep scratches. (**b**) Mask with only shallow scratches. (**c**) Mask containing two deep scratches and one shallow scratch.

The synthetic images should closely resemble the real scratch images and include scratches of varying depths to the greatest extent possible. To demonstrate the effectiveness of the MSDS, we compared it with the representative CutPaste method and the state-of-the-art (SOTA) industrial defect detection data synthesis method, SDAS. To visually illustrate the data synthesis performance of these methods, we created a set of masks from the labels in the test set and used them to generate images, with the results shown in Figure 6.

The MSDS employs a scratch fine-tuning process during mask generation to produce scratches that are deeper in the center and shallower at the edges, aligning with real-world scratch patterns. While CutPaste and SDAS excel in generating deep scratches in Figure 6a, their pattern-adding approach falters in Figure 6b,c for the shallow- and mixed-depth scratches, respectively. In these cases, appearance of the scratch is largely dictated by pattern differences rather than mask control, resulting in the overly deep shallow scratches in Figure 6b and indistinct depth variations in Figure 6c. In addition, this approach produces unrealistic anomaly patterns, as shown in the boxes (1), (2), and (4) in Figure 6. In contrast, MSDS leverages the mask-guided diffusion model to directly generate scratch images, controlling mean shift via mask depth values to achieve precise scratch depth variation. This direct generation improves image consistency and visual coherence. Consequently, MSDS enables the creation of a more balanced and comprehensive scratch dataset, which improves robustness and accuracy in the detection of scratches of varying depths.

### 3.4. Scratch Segment Results with TransCNN

To validate the authenticity and effectiveness of the MSDS-generated data, we followed the methodology in Section 3.2 to construct a training dataset using 1000 masks and trained TransCNN for scratch segmentation, evaluating its performance. To further assess TransCNN’s efficacy in scratch segmentation, we trained SegNet, U-Net, and SegFormer on the MSDS-generated dataset and compared their test results. We adopted the mean Intersection over Union (mIoU) and Dice coefficient as evaluation metrics, with IoU emphasizing scratch segmentation accuracy unaffected by background pixels and the Dice coefficient balancing precision and recall.

**Figure 7:**
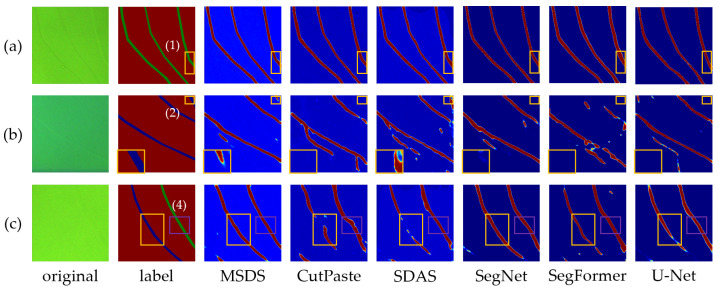
Heatmap of segmentation results for different methods. Labels: Red for background, green for deep scratches, and blue for shallow scratches. (**a**) Mask with only deep scratches. (**b**) Mask with only shallow scratches. (**c**) Mask containing one deep scratch and one shallow scratch.

**Figure 8:**
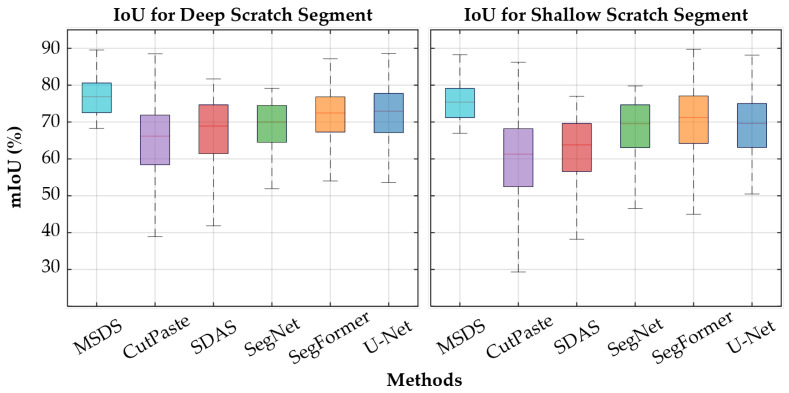
Box plots of the mIoU for different methods on deep and shallow scratches.

**The effectiveness of MSDS.** All methods are capable of realistically synthesizing deep scratches due to their distinct features, allowing trained models to effectively detect deep scratches. As illustrated in Figure 7a, all three methods successfully segment deep-scratch regions, achieving high mIoU and Dice coefficients, as detailed in Table 1. For shallow scratches, the MSDS, by directly generating scratches, produces a dataset that closely mirrors real-world conditions, outperforming CutPaste and SDAS in segmentation performance. As shown in Table 1, MSDS shows a greater advantage, surpassing SDAS by 19.8% in mIoU, while maintaining consistent performance relative to deep-scratch detection as illustrated in Figure 8. This is mainly due to the mask-guided generation of images with various scratch depths, resulting in a more comprehensive dataset and a model with enhanced generalization.

**The effectiveness of TransCNN.** TransCNN exhibits robust scratch segmentation performance. Compared to mainstream segmentation networks, its advantage in deep-scratch detection is modest, but it significantly outperforms SegNet in shallow scratches. As illustrated in Table 1, against the structurally similar U-Net, TransCNN achieves an 8.8% higher mIoU score, and it exceeds SegFormer, which shares a similar modular design, by 0.048 in mIoU. We attribute TransCNN’s superior performance over SegFormer and U-Net to its use of ViT blocks for feature encoding, which expands the receptive field and enhances global feature perception, particularly for smaller scratches. In addition, skip connections and CNN blocks enable spatial detail recovery for ViT-encoded features, resulting in improved segmentation accuracy, as shown in Figure 7.

### 3.5. Ablation Study

**Figure 9:**
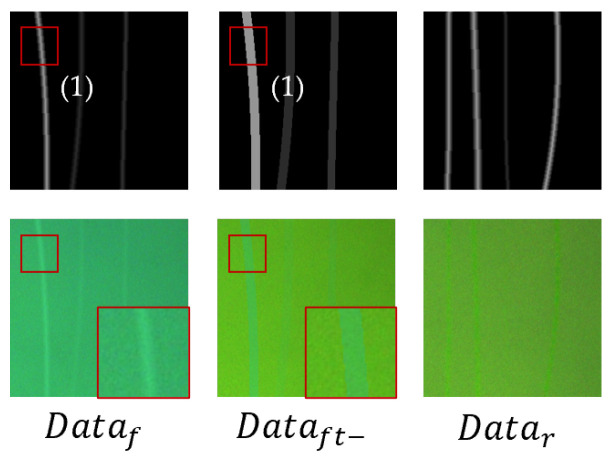
Scratch generation results under different mask sampling methods.

U-Net can serve as a control for the ablation study of the ViT encoding block due to its convolutional encoder-decoder architecture with skip connections. SegFormer, a ViT-based network, serves as a control for the ablation study of the skip-connected convolutional decoding structure. The results of these experiments have already been presented in Section 3.4 and are not repeated here. This section focuses on analyzing the factors affecting the performance of the MSDS, with results and metrics presented in Figure 9 and Table 2.

**Effect of $P\left(S\right)$.** To study the effect of sampling method on the generation of scratch images, we chose a completely random-sampling mask sampling method for comparison. A scratch dataset guided by masks generated by random sampling was created to train the TransCNN. We labeled the dataset sampled using $P\left(S\right)$ as $Data_{f}$ and the dataset sampled using random sampling as $Data_{r}$. The results are shown in Figure 9. Among them, the scratch width is too wide or too narrow in the $Data_{r}$, while the sampled scratch width is more uniform and more in line with the distribution of $P\left(S\right)$, thus, as illustrated in Table 2, a better scratch segmentation model can be trained.

**Effect of Scratch Fine-Tuning Process.** To investigate the impact of the FT process on scratch generation, we removed it from the complete sampling pipeline and repeated the experimental steps outlined in the previous section. We labeled the dataset sampled without the FT process as $Data_{ft-}$ and present a selection of results in Figure 9. Removing the FT process resulted in sharper edges in the generated images. Conversely, incorporating the FT process yielded more natural edge transitions, more closely resembling real-world scratches, and, consequently, leading to superior performance metrics.

## 4. Conclusions

A novel method for generating training data pairs was proposed, using a mask as an anomaly overlay to guide a diffusion model trained only on normal data. This approach enables the model to perform a local mean shift, creating realistic images with anomalies. The tailored TransCNN model, integrating ViT blocks to capture anomalous features and a convolutional decoder for spatial reconstruction, proved effective and industrially viable for chip scratch detection.

However, in real-world production lines, variations in lighting and camera angles can cause illumination unevenness or specular reflections, deviating from the image distribution modeled by MSDS and reducing the framework’s reliability. Future work will focus on developing robust anomaly synthesis techniques to handle complex environmental variations and extend the framework to diverse defect types, aiming for generalizable solutions in industrial inspection with limited labeled data.

## Figures and Tables

**Table 1:** Performance metrics of different methods for segmentation of deep and shallow scratches.

Method	mIoU (Deviation)		Dice (Deviation)	
	Shallow	Deep	Shallow	Deep
Ours	74.4 (7.5)	75.8 (7.6)	85.3 (6.1)	86.2 (6.0)
CutPaste	59.7 (10.9)	64.3 (10.1)	74.8 (9.2)	78.3 (8.3)
SDAS	62.1 (10.0)	66.9 (10.5)	76.6 (8.5)	80.2 (8.5)
SegNet	67.7 (9.8)	68.1 (9.9)	80.7 (8.0)	81.0 (8.4)
SegFormer	69.6 (10.1)	70.8 (9.4)	82.1 (8.0)	82.9 (7.7)
U-Net	68.4 (9.4)	71.3 (9.4)	81.2 (7.6)	83.2 (7.6)

**Table 2:** The impact of different modules.

Mark	Module		mIoU (%)		Dice (%)	
	Sampler	FT Process	Shallow	Deep	Shallow	Deep
$Data_{f}$	$P\left(S\right)$	Yes	74.4	75.8	85.3	86.2
$Data_{r}$	Random	Yes	65.9	67.9	79.3	80.9
$Data_{ft-}$	$P\left(S\right)$	No	68.7	71.4	81.4	83.3

## Data Availability

The data presented in this study are not publicly available due to privacy and ethical restrictions, as they involve sensitive core product data from a collaborating third-party inspection company.
